# Construction of a Robust *Sphingomonas* sp. Strain for Welan Gum Production via the Expression of Global Transcriptional Regulator IrrE

**DOI:** 10.3389/fbioe.2020.00674

**Published:** 2020-06-30

**Authors:** Xiaoliu Liu, Ming Zhao, Zheng Xu, Hong Xu, Sha Li

**Affiliations:** ^1^State Key Laboratory of Materials-Oriented Chemical Engineering, Nanjing, China; ^2^College of Bioscience and Engineering, Hebei University of Economics and Business, Shijiazhuang, China; ^3^College of Food Science and Light Industry, Nanjing Tech University, Nanjing, China; ^4^Jiangsu National Synergetic Innovation Center for Advanced Materials (SICAM), Nanjing, China

**Keywords:** welan gum, IrrE, robust, carotenoid free, RNA-seq

## Abstract

Welan gum is a widely used microbial polysaccharide produced by *Sphingomonas* sp. However, an important factor hindering the expansion of its production is the maladaptation of strain to fermentation conditions. In this work, the global transcriptional regulator gene *irre* was selected as a stress-resistant element. And it was integrated into the site of the genomic carotene synthesis key enzyme gene *crtB* to construct a robust carotenoid-free welan gum producing strain. Fermentation with the recombinant strain effectively reduced the ethanol consumption and pigment content in the product. The tolerance temperature increased by 10°C without the need for controlling the pH. Under this fermentation condition, welan gum concentration could still reach 20.26 ± 0.25 g/L, which was 187.38% higher than that of the wild-type strain (7.05 ± 0.15 g/L). Transcriptome analysis showed that with the control of IrrE, more than 1000 genes that are involved in multiple pathways, including two-component system, bacterial chemotaxis, flagellar assembly, and cell cycle, exhibited changes at the transcriptional level and jointly allowed the strain to protect against environmental stresses.

## Introduction

*Sphingomonas* sp. is a promising producer of gellan gum polysaccharide, such as gellan gum, welan gum, rhamsan gum, and so on. Welan gum is an important member of gellan gum family that is widely used in food, medicine, concrete additives, and oil recovery because of its high and stable viscosity in aqueous solution over a broad range of temperature and pH (Zhu et al., 2014a; Ai et al., 2015; Liu et al., 2017). However, many restrictive factors in gellan gum polysaccharide production limit its yield and large-scale application.

The main reason lies in the poor environment tolerance of the *Sphingomonas* sp. strain. For welan gum production, heat from mechanical agitation and microbial metabolism (Zhu et al., 2014a), high viscosity and weak acidity from welan gum accumulation (Li et al., 2011a), and pigment from carotenoid synthesis (Zhang et al., 2016) result in the large demand of cooling water, alkali binding agent, strong ventilation or agitation for fermentation, and alcohol for polysaccharide purification, thereby causing high energy consumption and cost.

Robustness toward industrial conditions is a key feature in engineering microorganisms for industrial metabolite production (Marc et al., 2017). For the past few years, mutation breeding, adaptive laboratory evolution, and resequencing experiments have been used to obtain advanced industrial strains (Barrick and Lenski, 2013). Zhu et al. (2014a) obtained a mutant *S*. sp. by atmospheric and room-temperature plasma-induced mutation, and the mutant could grow well at 37°C. Nevertheless, these approaches have difficult in mutation direction grasping, beneficial combinatorial landscape occurrence, target strain screening, and good traits passing on (Blooma and Arnoldb, 2009). Synthetic biological technologies, especially metabolic engineering, are used to improve the performance of industrial microorganisms (Chae et al., 2017). However, most cellular phenotypes are controlled by several genes. Current metabolic engineering approaches are limited by multiple gene modification (Alper et al., 2005).

Therefore, approaches such as generating global transcriptional regulators and heat shock proteins to achieve multiple regulatory have been developed (Lin et al., 2013; Mukhopadhyay, 2015). Transcriptional regulators play crucial roles in microbial growth and metabolism by regulating gene expression, repairing DNA or protein, and promoting the formation of biofilms (Alper and Stephanopoulos, 2007; Zhu et al., 2016). For example, mutational housekeeping σ^70^ factor (Gruber and Gross, 2003) enhances the ethanol and SDS tolerance of *Escherichia coli* (Santos and Stephanopoulos, 2008). Over-expressed transcriptional activator encoding gene *HAA1* increases the acetic acid tolerance of *Saccharomyces cerevisiae* (Tanaka et al., 2012). Exogenous DNA repair-related protein IrrE improves the radiation (Gao et al., 2003), osmosis, heat, and oxidation tolerance (Jie et al., 2009) of *E. coli* and the alcohol, heat, and osmosis tolerance of *Zymomonas mobilis* (Chen, 2010). Heat shock proteins are involved in important physiological processes, such as proper folding, aggregation, transport, and signaling of proteins (Lindquist, 1992; Yu et al., 2015). They are synthesized under stress conditions to guarantee cell survival. GroESL overexpression endows *E. coli* with a remarkable tolerance to n-butanol, i-butanol, 2-butanol, 1,2,4-butanetriol, and ethanol (Zingaro and Terry Papoutsakis, 2013) and imparts *Clostridium. tyrobutyricum* with tolerance to butyric acid (Suo et al., 2017). The heteroexpression of *Tamarix hispida* Hsp18.3 improves the resistance of yeast to heat, osmosis, and heavy metals (Gao et al., 2012). Nevertheless, no strategy has been developed to increase the resistance of welan gum-producing strains.

In addition to poor resistance to environmental stress, the synthesis of carotenoids accompanying welan gum production is another issue that should be considered. Carotenoids are derived from the isoprenoid or steroidal pathway with isopentenyl pyrophosphate (IPP) as a precursor. In fungi, archaea, and most eukaryotes, IPP is synthesized by acetyl CoA via the mevalonate pathway. Meanwhile, in most bacteria and algae, IPP is produced by pyruvate and glyceraldehyde-3-phosphate via the methyl erythritol pathway (Armstrong et al., 1990; Preejith et al., 1996). Wu et al. analyzed the carotenoid synthesis pathway in the gellen gum-producing strain *Sphingomonas eloda* ATCC31461. The key enzymes of this strain include phytoene synthase, phytoene dehydrogenase, lycopene cyclase, 2,2’-β-ionone ring hydroxylase, and 3,3’-β- ionone ring hydroxylase, and the coding genes are *crtB*, *crtI*, *crtY*, *crtG*, and *crtZ*, respectively (Wu et al., 2011). Similar results were also confirmed in another welan gum-producing strain, *Sphingomonas* sp. ATCC31555 (Zhang et al., 2016). These results provided a useful reference for our research.

In the present work, heat shock proteins and global transcriptional regular factor from extremophile were expressed in the welan gum producing-strain *S*. sp. NX-3 to enhance its complex phenotypes. The selected stress-resistant element was then inserted into the key enzyme gene site of the carotene synthesis pathway in the genome, thereby constructing a pigment production defect strain with multiplex stress resistant. Subsequently, fermentation performance and genome-wide transcriptional analyses were conducted to investigate the external and internal influences of the metabolic modification above. Although we focused on the robustness of the welan gum-producing strain, the concepts discussed herein are also applicable to other industrial strains.

## Materials and Methods

### Strains and Plasmids

Relevant characteristics of plasmids and strains are listed in Table 1. *E. coli* DH5α harboring recombinant pBBR1MCS-5 plasmids and *E. coli.* S17-1 harboring recombinant pJQ200SK plasmids were used as the donors in conjugal transfer. *E. coli* HB101 (pRK600) was served as the helper strain in conjugal transfer. And, *S*. sp. NX-3 and its derivatives were employed as the acceptors in this study.

**TABLE 1:** Strains and plasmids used in this study.

Strains or plasmids	Characteristics	Sources
**Strains**		
*Thermoanaerobacter tengcongensis* CGMCC5161	Source of resistance *groes* gene	This lab
*Thermus thermophilus* CGMCC15059	Source of resistance *groel* gene	This lab
*Deinococcus radiodurans* CGMCC633	Source of resistance *irre* gene	This lab
*E. coli* DH5α	Host strain for cloning vectors	TaKaRa
*E. coli-*pET-*groes*	*E. coli* DH5α harboring pET-*groes*; Km^*r*^	This work
*E. coli-*pET-*groel*	*E. coli* DH5α harboring pET-*groel*; Km^*r*^	This work
*E. coli-*pET-*irre*	*E. coli* DH5α harboring pET-*irre*; Km^*r*^	This work
*E. coli-*pBBR-*groes*	*E. coli* DH5α harboring pBBR-*groes*; Gm^*r*^	This work
*E. coli-*pBBR-*groel*	*E. coli* DH5α harboring pBBR-*groel*; Gm^*r*^	This work
*E. coli-*pBBR-*irre*	*E. coli* DH5α harboring pBBR-*irre*; Gm^*r*^	This work
*E. coli* HB101(pRK600)	Conjugation helper strain	provided by Nanjing Agricultural University
*S.* sp. NX-3	Welan gum producing strain; Sm^*r*^	This lab
*S.* sp.-pBBR-*groes*	*S.* sp. NX-3 harboring pBBR-*groes*; Sm^*r*^, Gm^*r*^	This work
*S.* sp.-pBBR-*groel*	*S.* sp. NX-3 harboring pBBR-*groel*; Sm^*r*^, Gm^*r*^	This work
*S.* sp.-pBBR-*irre*	*S.* sp. NX-3 harboring pBBR-*irre*; Sm^*r*^, Gm^*r*^	This work
*E. coli* S17-1	Host strain for suicide vectors	provided by Nanjing Agricultural University
*E. coli* S17-pJQ-Δ*crtB*	*E. coli* S17-1 harboring pJQ-Δ*crtB*	This work
*S.* sp.*-*Δ*crtB*	*S.* sp. NX-3 with *crtB* knockout	This work
*S.* sp.*-*Δ*crtB-irre(named S.* sp. NX-R)	*S.* sp. NX-3 with *crtB* knockout and *irre* insertion	This work
**Plasmids**		
pET28a	Expression vector; Km^*r*^	TaKaRa
pET-*groes*	pET28a harboring *groes* gene; Km^*r*^	This work
pET-*groel*	pET28a harboring *groel* gene; Km^*r*^	This work
pET-*irre*	pET28a harboring *irre* gene; Km^*r*^	This work
pBBR1MCS-5	Broad-host-range cloning vector; Gm^*r*^	provided by Nanjing Agricultural University
pBBR-*groes*	pBBR1MCS-5 harboring *groes* gene; Gm^*r*^	This work
pBBR-*groel*	pBBR1MCS-5 harboring *groel* gene; Gm^*r*^	This work
pBBR-*irre*	pBBR1MCS-5 harboring *irre* gene; Gm^*r*^	This work
pJQ200SK	suicide vector; Gm^*r*^	provided by Nanjing Agricultural University
pJQ-Δ*crtB*	*crtB* gene deletion vector; Gm^*r*^	This work
pJQ-Δ*crtB-irre*	*irre* gene insertion vector; Gm^*r*^	This work

#### Recombinant Plasmids Construction

In-fusion cloning was used to construct the recombinant plasmids. The primers used in this study are listed in Supplementary Table S1.

For the construction of the recombinant *Sphingomonas* expression vector pBBR-*groes*, the gene *groes* (Sequence ID: AE008691.1) carrying homologous arms on both ends was amplified using the primer pair pET-*groes*-F/pET-*groes*-R with the genome of *Thermoanaerobacter tengcongensis* MB4 as a template. After purification and sequencing, the fragment above was cloned into linearized vector pET28a with the restriction sites of *Nco* I and *Not* I, forming plasmid pET-*groes*. The recombinant pET-*groes* plasmid was used as a template to amplify the gene *groes* carrying the T7 promoter, which was purified, sequenced, and ligated into the linearized broad host plasmid pBBR1MCS-5 with *Hind* III/*BamH* I to obtain recombinant *Sphingomonas* expression vector pBBR-*groes*. The plasmid construction and gene deletion processes are shown in Supplementary Figures S1, S2. This method is also applicable to the construction of pBBR-*groel* (Sequence ID for *groel was* AP008226.1) and pBBR-*irre* (Sequence ID for *irre was* CP015081.1).

For the construction of the *crtB* gene deletion vector, the upstream and downstream flanking sequences of the *crtB* gene were amplified using the degenerate primer pairs *crtB*-LF/*crtB*-LR and *crtB*-RF/*crtB*-RR. The two fragments were joined by overlap-PCR and cloned into the lined suicide vector pJQ200SK (*BamH* I/*Xba* I), resulting in the *crtB* gene deletion vector pJQ-Δ*crtB*. This method is also applicable to the construction of *irre* gene insertion vector pJQ-Δ*crtB*-*irre*.

#### Conjugation Transfer

Strains and plasmids used in this study were shown in the Table 1. Recombinant *Sphingomonas* expression vectors and *crtB* gene deletion vector pJQ-Δ*crtB* were transformed into *E. coli* DH5α and *E. coli* S17-1, respectively, yielded the donors in conjugal transfer. The specific process of conjugation transfer method was described in the previous study (Liu et al., 2017). The colonies that developed on the screening medium were candidates of *S*. sp. harboring expression vectors. Two steps of homologous recombination were needed to obtain the *crtB* knockout and *irre* insertion strains. Thus, the colonies that developed on the screening medium should be cultured in seed medium for *Sphingomonas* containing 5% (v/v) sucrose overnight and the culture solution was then diluted and spread at the screening medium containing 5% (v/v) for the selection of colorless clones.

### Media and Culture Conditions

LB medium (g/L): peptone 10, yeast powder 5, NaCl 10 (solid plus 1.5% agar). The final concentration of each antibiotic in the *E. coli* resistant medium was (μg/mL): kanamycin 25, gentamicin 50.

Agar medium for *S.* sp. (g/L): glucose, 10; beef extract, 3; peptone, 10; NaCl, 5; and agar, 20, pH 7.2–7.4. Seed medium for *S.* sp. (g/L): glucose, 20; yeast extract, 1; peptone, 3; K_2_HPO_4_⋅3H_2_O, 2; and MgSO_4_, 0.1, pH 7.2–7.4. Fermentation medium for *S.* sp. (g/L): glucose, 50; yeast extract, 8; K_2_HPO_4_⋅3H_2_O, 3; and MgSO_4_, 0.4, pH7.2–7.4 (Zhu et al., 2014a, b). Conjugative transfer screening medium (g/L): glucose 20, yeast extract 1, peptone 3, K_2_HPO_4_⋅3H_2_O 2, MgSO_4_ 0.1, pH 7.2–7.4. Final concentration of each antibiotic (μg/mL): gentamicin 50, streptomycin 100. Final concentration of isopropyl-β-d-thiogalactoside (μg/mL): 0.4, and added at the initial stage of fermentation.

For flask cultivation, strains were streaked out from frozen glycerol stocks onto plates containing the activation medium and incubated at 30°C for 36 h. One loop of colonies was inoculated in 100 mL seed medium in a 500 mL flask. After 16h, the seed culture was inoculated into 100 mL fermentation medium in a 500 mL flask, and the initial optical density at 600 nm (OD_600_) was adjusted to 0.2. The culture was incubated at 30°C and 200 rpm for 66 h.

For fermentor cultivation, 100 mL seed culture was first prepared in 500 mL flasks at 30°C for 16 h, then it was transferred to a 7.5 L fermentor (New Brunswick Scientific BioFlo 110, United States) containing 4.5 L fermentation medium, and the pH was adjusted to 7.4 with 3 M NaOH and the media were autoclaved prior to use. The aeration rate was maintained at 1.0 vvm with an agitation speed of 600 rpm for 60 h. To test the effect of the stress-resistant elements, different temperature gradients between 30 and 43°C, and different pH gradients between 4 and 10 might be used.

### Analytical Methods

Dry cell weight (DCW) was measured to reflect the cell growth, which was determined as previously reported (Li et al., 2011a). Glucose content was measured using a biosensor equipped with a glucose oxidase electrode (SBA-40C, Shandong Academy of Sciences, China). Culture broth viscosity and rheological behavior of welan gum solutions were measured by a rotational viscometer (NDJ-1, Shanghai Hengping Scientific Instrument Company, China) with rotor No. 4 at 60 rpm (Ai et al., 2015; Li et al., 2011b). The welan gum production was determined by isopropanol precipitation and dry weight measurement (Li et al., 2010).

### RNA-Seq Analysis

Total RNA was extracted in biological triplicates from strains *S*. sp. NX-3 and *S*. sp. NX-R cultured at 30°C and pH 7.0 and *S*. sp. NX-R cultured at 40°C and natural pH for 20 h using TaKaRa RNAiso Plus kit. The concentration and purity of total RNA was determined using NanoDrop, and the integrity of the RNA was identified using Agilent 2100 and simulated electrophoresis gel images. The qualified RNA samples were sent to the Beijing Genomics Institute (BGI) for library constructing and sequencing. After data filtering, reference genome comparison and gene expression analysis, significant differentially expressed genes were filtered for genes with log2 fold change > 1 (upregulation) or log2 fold change < -1 (downregulation) with *p*-value and FDR both <0.05 (Tang et al., 2008; Xie et al., 2011). Significant differentially expressed genes were further cluster analyzed and functional enrichment analyzed to determine its most important biochemical metabolic pathways and signal transduction pathways.

## Results

### Effects of Stress-Resistant Elements on the Tolerance of *S*. sp. Against Thermal Shock

Firstly, stress-resistant elements derived from extremophiles, including heat shock proteins GroES of *Thermoanaerobacter tengcongensi*, GroEL of *Thermus thermophilus* and global transcriptional regulator IrrE of *Deinococcus radiodurans*, were heterologously expressed in the welan gum-producing strain *S*. sp. NX-3 to improve cell tolerance.

**FIGURE 1:**
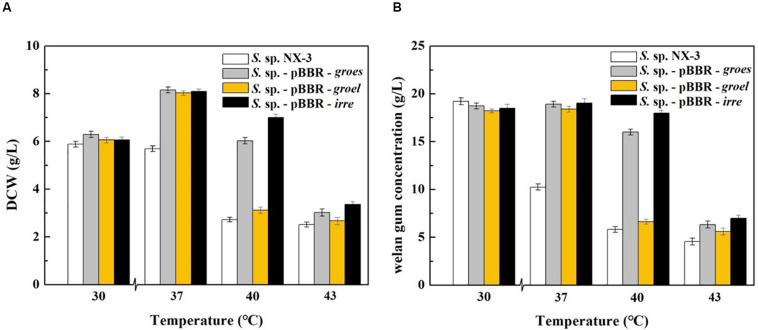
Effects of stress-resistant elements on the tolerance of *S*. sp. against thermal shock. **(A)** Effects of stress-resistant elements on DCW at different temperatures. **(B)** Effects of stress-resistant elements on welan gum concentration at different temperatures.

With the increase in fermentation temperature from 30°C to 43°C, both the final DCW and welan gum concentration of the control strain decreased (Figure 1). The maximum biomass and welan gum concentration at 30°C were 5.89 ± 0.12 g/L and 19.23 ± 0.20 g/L, respectively. Meanwhile, all of the recombination strains showed a good performance at 37°C not only in maximum biomass but also in maximum welan gum concentration. At 30°C, the difference between the recombinant and control strains in terms of bacterial biomass and welan gum concentration was not significant. When the fermentation temperature increased to 43°C, the cell growth of *S*. sp. NX-3 was almost stagnant, followed by the cessation of welan gum synthesis. The expression of the stress-resistant elements did not have significant positive effects to the results. When the fermentation temperature increased to 40°C, the recombinant strains *S*. sp.-pBBR-*groes* and *S*. sp.-pBBR-*irre* showed significant advantage compared with the control strains, although the biomass and welan gum concentration at the end of fermentation process were slightly lower than those of 37°C. Recombinant strain harboring global transcriptional regulator IrrE performed well, with the DCW and welan gum concentration reaching 7.00 ± 0.11 g/L and 17.98 ± 0.28 g/L, thereby obtaining 1.57- and 2.09-fold increase than those of the control strains (2.72 ± 0.10 g/L and 5.82 ± 0.11 g/L), respectively.

### Effects of Stress-Resistant Elements on the Tolerance of *S.* sp. Against Weak Acidity Shock

**FIGURE 2:**
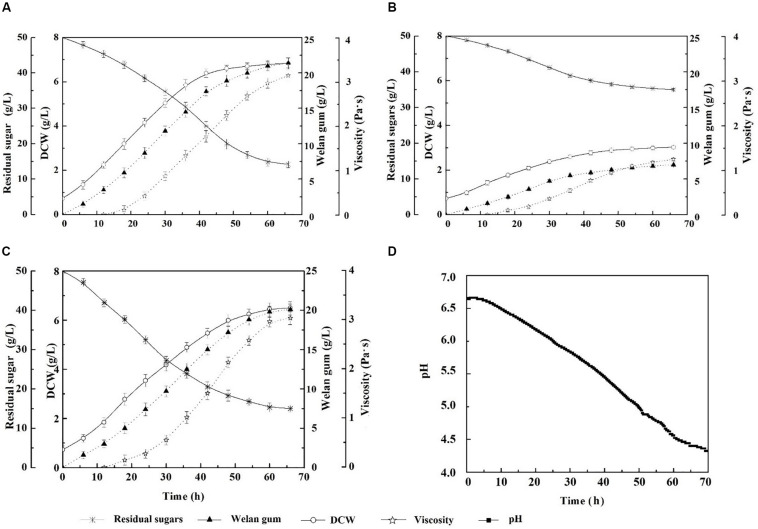
Effects of stress-resistant elements on the tolerance of *S*. sp. against weak acidity shock. **(A)** Time profiles of *S*. sp. NX-3 fermented at 30°C, and pH range of 7.2–7.4. **(B)** Time profiles of *S*. sp. NX-3 fermented at 40°C and nature pH. **(C)** Time profiles of *S*. sp.-pBBR-*irre* fermented at 40°C and nature pH. **(D)** Time profiles of pH changes during *S*. sp.-pBBR-*irre* fermentation at 40°C.

The growth and welan gun production of the reconstruction strain expressing IrrE under natural pH conditions at this temperature were investigated. The results were shown in Figure 2. As the fermentation process progressed, the acidic polysaccharide gradually accumulated, and the final pH of the fermentation broth decreased to approximately 4.2. Under this condition, the DCW of *S*. sp.-pBBR-*irre* was 6.50 ± 0.12 g/L, which was equal to 95.17% of the outcome of the wild-type strain at optimum fermentation conditions (30°C and pH of 7.0). Correspondingly, the welan gum concentration of the IrrE expressing strain was 20.12 ± 0.20 g/L, which reached 94.02% of that of the wild-type strain at optimum conditions (21.40 ± 0.30 g/L). Meanwhile, the DCW and welan gum concentration of *S.* sp. NX-3 were 3.02 ± 0.11 g/L and 7.00 ± 0.20 g/L, which were only 46.46% and 34.79% of those of the IrrE expressing strain, respectively. The expression of the global regulatory factor IrrE remarkably improved the resistance of *S*. sp. to acidic pH.

In summary, *S*. sp.-pBBR-*irre* is suitable for the fermentation process at 40°C and natural pH conditions, which can effectively decrease the amount of cooling water, remove the use of neutralizing alkali and pH electrode, simplify the fermentation device, and reduce the production cost.

### Construction of Carotenoid-Free Welan Gum-Producing Strain

Comparative genomics was used to evaluate the key genes involved in carotenoids synthesis of in *S*. sp. NX-3. Compared with previous studies, *S.* sp. NX-3 had extremely high homology with the gellan gum-producing strain *Sphingomonas* elodea ATCC31461 and another welan gum-producing strain *Sphingomonas* sp. ATCC31555 (95% and 98%, respectively) (Gai et al., 2011; Wang et al., 2012), whose pigment synthesis pathways have been reported. Genes *crtB*, *crtI, crtY*, *crtG*, and *crtZ* were amplified using the *S*. sp. NX-3 genome as a template. After sequencing, the gene sequences of 923, 1478, 797, 1157, and 501 bp were obtained, which were consistent with the prospective length. Through BLAST alignment, the homology between the genes in *S*. sp. NX-3 and *S*. *elodea* ATCC31461 and *S*. sp. ATCC31555 reached 99%. Therefore, *S*. sp. NX-3 may have the same key genes for pigment synthesis.

**FIGURE 3:**
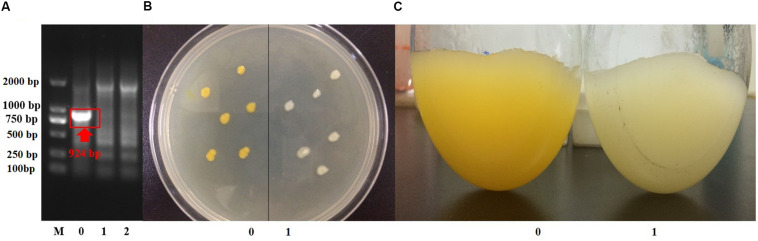
Confirmation of *crtB* deletion. **(A)** Confirmation of *crtB* deletion by PCR using primer pairs of crtB-F/crtB-R with chromosomal DNA serving as the template for amplification. Lane M, DNA marker; lane 0, wild-type strain amplified; lanes 1 and 2, gene deletion strains amplified. **(B)** Comparison of the phenotypes of wild-type and gene deletion strains. 0, wild-type strains; 1, gene deletion strains. **(C)** Comparison of the morphologies of wild-type and gene deletion strains. 0, wild-type strain; 1, wild-type strain.

Suicide vector with the up- and downstream homologous arms of *crtB* was constructed, and strain *S*. sp.-Δ*crtB* was obtained after conjugation transfer and homologous recombination (Figure 3A). The original (Figure 3B-0) and *crtB* deletion bacteria (Figure 3B-1) were inoculated onto the same plate to compare the colonial morphology. As shown in the figure, before and after the *crtB* gene knockout, the colonial morphology did not change significantly, both were round, and the edges were smooth. However, after *crtB* gene deletion, the colonies appeared colorless. The shake flask fermentation results (Figure 3C) also showed that the fermentation broth turned white after the *crtB* gene was knocked out. This result further indicated that the *crtB* gene is one of the key genes of the pigment synthesis pathway in *S*. sp. NX-3, and knocking *crtB* gene can effectively block the production of yellow pigment in *S*. sp.

Welan gum production at 30°C and pH of 7.2–7.4 and ethanol consumption before and after *crtB* deletion are shown in Table 2. The welan gum concentration of *S*. sp.-Δ*crtB* was slightly higher than that of *S*. sp. NX-3, possibly because *crtB* knockout blocked the synthesis of carotenoids and made the more metabolic flow turned to the welan gum synthesis pathway. Ethanol consumption, ethanol cost, and product color were also compared. The results indicated that *crtB* deletion can reduce the ethanol cost by 36.9% in the purification process, and the product color was significantly better than the product before knockout. Lower pigment content in waste ethanol was also beneficial to its reuse.

**TABLE 2:** Effects of *crtB* deletion on welan gum production and purification.

Parameters	Strains	
	
	*S*. sp. NX-3	*S*. sp.-Δ *crtB*
Welan gum concentration (g/L) ^*a*^	21.34 ± 0.20	22.54 ± 0.20
Ethanol consumption (L/L fermentation broth)	3	2
Ethanol cost ($/kg dry welan gum) ^*b*^	85.75	54.12
Product color	Light yellow	White

*^*a*^See Figure 5C, fermentation conditions was 30°C, pH7.2–7.4. ^*b*^ Elcohol’s price ($/kg dry welan gum) = $\frac{n\times 1000}{c}\times P$, where n- is the ethanol consumption (L/L fermentation broth), c- is the welan gum concentration (g/L), and P- is the unit price of ethanol ($/t, $569.00/t0. Ethanol reuse was excluded. Thus, the calculation is higher than the actual cost.*

### Construction of Multi-Resistance Carotenoid-Free Welan Gum-Producing Strain

**FIGURE 4:**
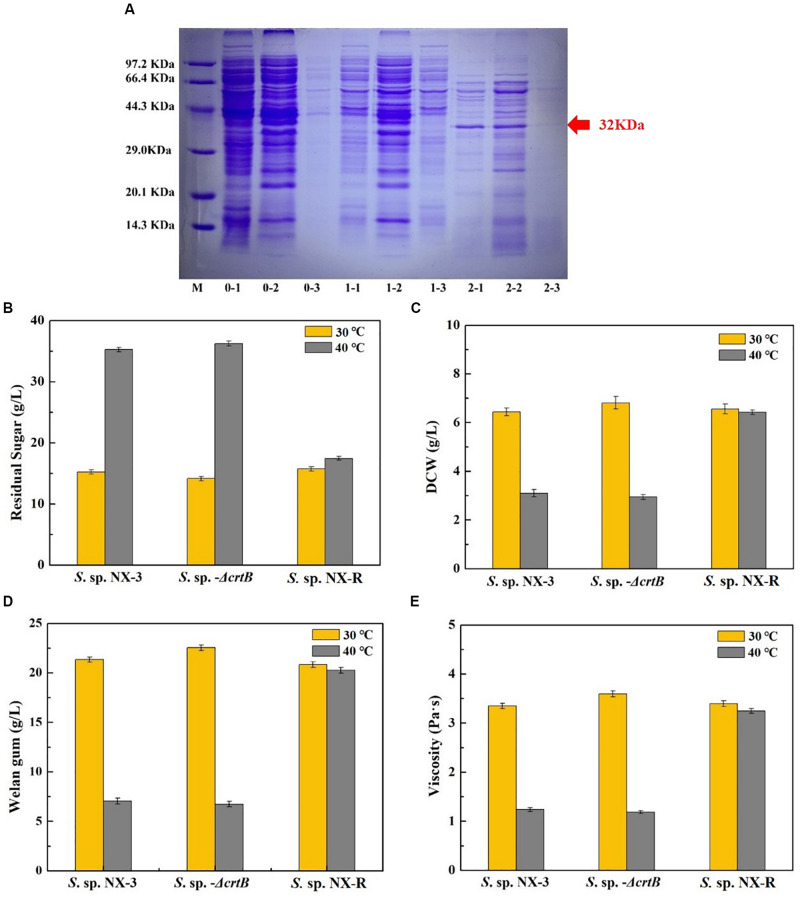
Confirmation of IrrE expression and its effect on welan gum fermentation at different temperatures and nature pH. **(A)** Identification of IrrE expression by SDS-PAGE. M, unstained protein marker; lanes 0-1, whole cells of wild-type strain; lanes 0-2, supernatant after ultrasonication of wild-type strain; lanes 0-3, precipitate after ultrasonication of wild-type strain; lanes 1-1, whole cells of uninduced *irre* insertion strain; lanes 1-2, supernatant after ultrasonication of uninduced *irre* insertion strain; lanes 1-3, precipitate after ultrasonication of uninduced *irre* insertion strain; lanes 2-1, whole cells of IPTG induced *irre* insertion strain; lanes 1-2, supernatant after ultrasonication of IPTG-induced *irre* insertion strain; lanes 1-3, precipitate after ultrasonication of IPTG induced *irre* insertion strain. **(B)** Effects of IrrE expression on residual sugar at different temperatures. **(C)** Effects of IrrE expression on DCW at different temperatures. **(D)** Effects of IrrE expression on welan gum concentration at different temperatures. **(E)** Effects of IrrE expression on broth viscosity at different temperatures.

The results of See section “Effects of Stress-Resistant Elements on the Tolerance of *S*. sp. Against Thermal Shock” show that the expression of the global transcriptional regulator IrrE can effectively improve the tolerance of the strain to high temperature and acidic pH. However, the plasmid expression exhibited poor stability, and antibiotics required extremophiles, thereby resulting in high cost and disadvantages in later gene manipulation. To overcome these defects, *S.* sp. NX-R strain was constructed by inserting the *irre* gene into the *crtB* site of the *S.* sp. NX-3 genome, and a band with a molecular mass of approximately 32 kDa was visualized from recombinant strain after induction (Figure 4A). The fermentation of *S*. sp. NX-3, *S*. sp.-Δ*crtB*, and *S.* sp. NX-R was compared under different fermentation conditions. At 30°C and pH of 7.2–7.4, the differences in glucose consumption (Figure 4B), DCW (Figure 4C), welan gum concentration (Figure 4D), and broth viscosity (Figure 4E) among the three strains were not significant. The results revealed that the *crtB* deletion and *irre* integration had no considerable influence on the fermentation performance of the strains. While, when fermentation at 40°C, the four parameters above are lower than those at 30°C. However, the welan gum concentration of *S*. sp. NX-R can still reach 20.26 ± 0.25 g/L compared with that of 30°C (20.85 ± 0.20 g/L), with only 2.91% decrease. Compared with *S*. sp. NX-3 (7.05 ± 0.15 g/L), *S*. sp.-Δ*crtB* (6.74 ± 0.20 g/L), the welan gum concentration of *S.* sp. NX-R increased by 187.38% and 197.63%, respectively. In terms of dose, the expression of the global transcription factor IrrE can facilitate the tolerance of the welan gum-producing strain to abiotic stress and enhance its industrial application value.

### Genome-Wide Transcriptional Analysis of IrrE-Expressing Strain in Response to Abiotic Stresses

To understand how global transcription regulator IrrE may confer multiple tolerance to *S*. sp., we performed RNA-seq of three samples (*S*. sp. NX-R at 30°C VS *S*. sp. NX-3 at 30°C, and *S*. sp. NX-R at 40°C VS *S*. sp. NX-3 at 30°C) to analyze global changes in gene expression. Overall, the average output of each sample was 1.34 Gb, and the clean reads, which were directly used for subsequent analysis, accounted for more than 97% of the total reads.

**FIGURE 5:**
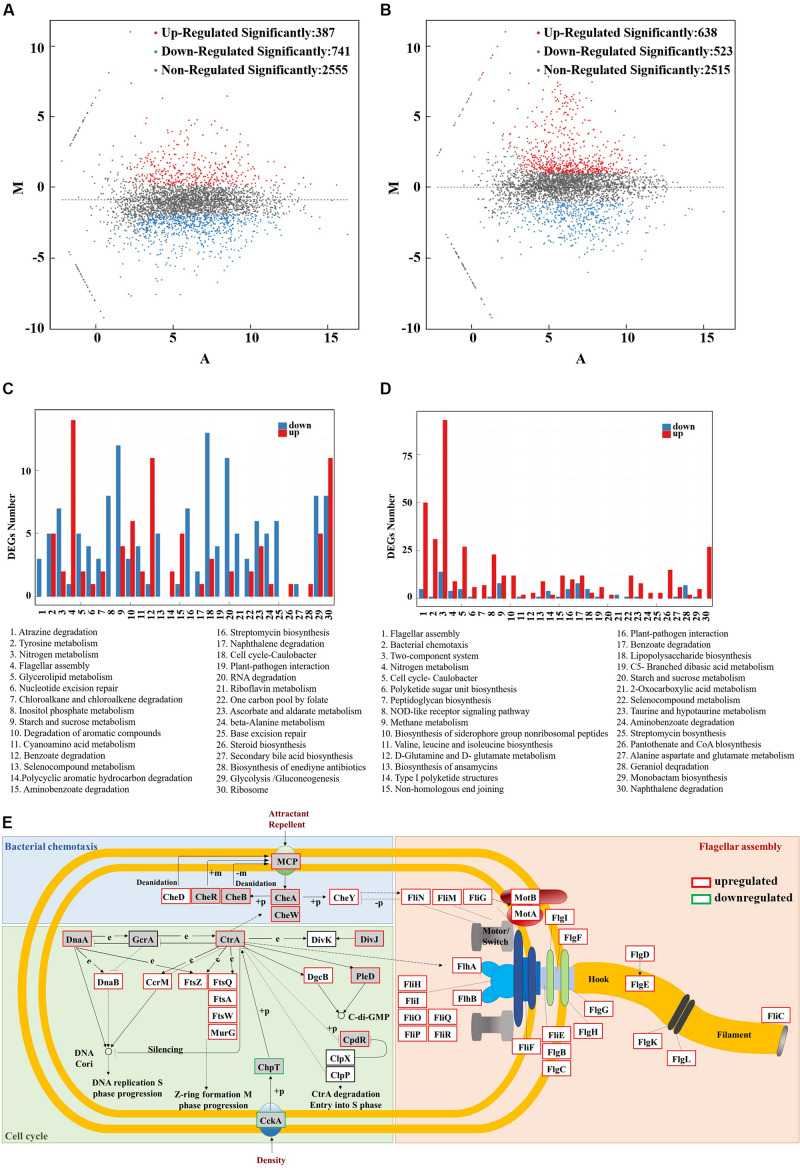
RNA-seq analysis of the IrrE-expressing strain in response to abiotic stresses. **(A)** MA plot of differentially expressed genes - *S*. sp. NX-R at 30°C VS *S*. sp. NX-3 at 30°C. **(B)** MA plot of differentially expressed genes - *S*. sp. NX-R at 40°C VS *S*. sp. NX-3 at 30°C. **(C)** Differentially expressed genes number of the most enriched pathway- *S*. sp. NX-R at 30°C VS *S*. sp. NX-3 at 30°C. **(D)** Differentially expressed genes number of the most enriched pathway- *S*. sp. NX-R at 40°C VS *S*. sp. NX-3 at 30°C. **(E)** Effect of IrrE expression on the transcriptional level of key genes in TCSs (In the gray box), flagellar assembly (in the orange box), bacterial chemotaxis (in the green box), and cell cycle (1n the blue box) at 40°C.

There are 387 significantly upregulated genes and 741 significantly downregulated genes in *S*. sp. NX-R compared with *S*. sp. NX-3 at 30°C (Figure 5A). Among them, the top 30 enriched pathways of the significantly different expressed genes were showed in the Figure 5C. The 5 most enriched pathways including atrazine degradation (ko00791), tyrosine metabolism (ko00350), nitrogen metabolism (ko00910), flagellar assembly (ko02040), glycerolipid metabolism (ko00561) etc., which are affiliated with the xenobiotics biodegradation and metabolism, amino acid metabolism, energy metabolism, cell motility, lipid metabolism of Kyoto Encyclopedia of Genes and Genomes (KEGG) pathway mapping. Interestingly, all Qvalues of the most enriched pathways were greater than 0.05. These results suggested that expression of *irre* gene may have very slight effects on cell growth and metabolism at 30°C.

Under abiotic conditions (40°C), 946 significantly upregulated genes and 490 significantly downregulated genes in multi-resistance strain were observed (Figure 5B). Compared with the results of *S*. sp. NX-R at 30°C VS *S*. sp. NX-3 at 30°C, flagellar assembly (ko02040), bacterial chemotaxis (ko02030), two-component system (TCSs, ko02020), and cell cycle (ko04112) become the most enriched pathways (Figure 5D). Then, the transcription level and locations of the significantly regulated genes involved in the pathways above are exhibited in the Figure 5E, and as is shown, most key genes in the four pathways are significantly upregulated. Here, TCSs, bacterial chemotaxis pathways and flagellar assembly pathways all play the functions of signal perception, conduction and cell motility. The bacterial methyl-accepting chemotaxis proteins (MCPs) sensed environmental signals and triggered a stimulatory response, which was transmitted to the flagellar motor switch protein FliN, FliM, FliG via sensor kinase CheA and chemotaxis protein CheY. The purine-binding chemotaxis protein CheW was also needed for the connection of CheA and MCPs, and chemotaxis protein methyltransferase CheR, protein-glutamate methylesterase/glutaminase CheB, chemotaxis protein CheD were needed for the modification of methylation and demethylation of MCPs to adapt to the environment. Correspondingly, the transcription levels of the flagellar biosynthesis proteins FlhA, FlhB, FliO, FliP, FliQ, and FliR, flagellar assembly protein FliH, flagellar basal-body rod modification protein FlgD, FlgE, FlgF, flagellar hook-associated protein FlgK, FlgK, and flagellar ring protein FliF, FlgI, FlgH, genes were also significantly upregulated.

In the cell cycle pathway, sensor histidine kinase/response regulator CckA, histidine phosphotransferase ChpT was downregulated, while cell cycle response regulator CtrA was upregulated. Meanwhile, the chromosomal replication initiator protein DnaA, and the replicative DNA helicase DnaB, the cell division protein FtsZ, FtsQ, FtsA, FtsW, the UDP-N-acetylglucosamine -N-acetylmuramyl-(pentapeptide) pyrophosphoryl-undecaprenol N-acetylglucosamine transferase MurG, actived by DnaA and CtrA, were all upregulated, so as to propel the DNA replication S phase progression and Z-ring formation M phase progression of the cell cycle. Thus, the cell proliferation was rapidly proceed to maintain the microbial population to cope with environmental challenges.

These results indicated that under the control of IrrE, the transcription level of key genes in the pathways of signal transduction, cell motility, cell growth and death, etc., changed significantly, so as to respond to the abiotic conditions and improve the stress resistance of microorganisms.

## Discussion

The high energy consumption and cost in welan gum production process is a challenging issue for the current approaches. In this work, we selected IrrE derived from extremophiles as a stress-resistant element to improve the tolerance of *S*. sp. against heat and weak acidity shock. We investigate key genes of carotenoid synthesis in *S.* sp. NX-3 and constructed pigment-free cells. Afterwards, a robust welan gum production strain by integrating IrrE into the key gene site of carotenoid synthesis was constructed. Finally, we investigated that IrrE plays as the global transcriptional regulatory factor in regulating the expression of genes in TCSs, bacterial chemotaxis, flagellar assembly, and cell cycle pathways, which jointly promote the survival of cells and enhance production capacity under non-physiological environmental conditions.

Many groups have attempted to manipulate transcriptional regulation and protein post-translational modification directly or indirectly by modifying transcriptional regulator factors and heat shock proteins, to improve strain resistance, respectively. And, most results were satisfactory. Nevertheless, long experiment period and low success rate caused by the ambiguity of the metabolic network and the complexity of gene manipulation of the non-model strain limited the application of large-scale metabolic engineering in *S*. sp. Thus, it is even more valuable to modify one or several genes to achieve multiple beneficial changes in cell phenotype. Here, expression of the selected global transcriptional regulatory factor IrrE overcame the above-mentioned problems, and, it effectively reduces the demand of cooling water, alkali binding agent, and purified alcohol in the production process.

Transcriptome analysis explained how strain to protect against abiotic stresses under the control of stress-resistant element IrrE. TCSs serve as a major stimulus-response coupling mechanism to allow organisms to sense and respond to changes in many different environmental conditions (Stock et al., 2000). TCSs controlled almost all bacterial physiological pathways, such as cell-to-cell signaling, chemotaxis, sporulation, osmolarity, nutrient assimilation, cell differentiation, and virulence (Stock et al., 2000). Bacterial chemotaxis and flagellar assembly were interacted with histidine kinases (CheA) and reflex regulators (CheY) of the TCSs to stimulate the microbial chemotactic motility to the external environment (West and Stock, 2001). The significant up-regulated of these genes motivated bacteria to “hasten after benefit and avoid damage.” The CckA-CtrA two-component signaling system was also plays an important role in the cell cycle pathway (Biondi et al., 2006). Density-dependent CckA kinase phosphorylates CtrA through the single domain histidine phosphotransferase, ChpT (Narayanan et al., 2018). And, the differential activity of CtrA is of paramount significance for generating different cell fates by regulating the cell division protein, modification methylase, and replicative DNA helicase directly or indirectly, thereby warrants the cells to replication in large numbers to keep the population alive under non-physiological environmental conditions (Westbye et al., 2018). Apart from working for cell cycle pathway, CtrA also serves as a transcription factor to drive the expression of key genes in the bacterial chemotaxis and flagellar assembly pathways. In general, under the role of the global transcriptional regulatory factor IrrE, different genes coordinated with each other to perform their biological functions and jointly promote the survival of cells and enhance production capacity under non-physiological environmental conditions.

## Conclusion

The present study aimed to solve the high energy consumption and cost in welan gum production process. The screened global transcriptional regulatory factor IrrE was integrated into the key gene site of carotenoid synthesis. Thus, a multi-resistance welan gum-producing strain with pigment synthesis defect was constructed. When fermented, the tolerance temperature can increase to 10°C without the need for pH control, and the final welan gum yield only had a slight decrease. Transcriptome analysis showed that under the control of stress-resistant element IrrE, more than 1000 genes, involved in multiple pathways, including two-component system, bacterial chemotaxis, flagellar assembly, biofilm formation, and cell cycle, etc., exhibited changes at the transcriptional level and allowed the strain to protect against abiotic stresses. The results will provide a useful reference of tolerance improvement for industrial microorganisms.

## Data Availability Statement

The datasets presented in this study can be found in online repositories. The names of the repositories and accession numbers can be found below: https://www.ncbi.nlm.nih.gov/, SRR11496385 https://www.ncbi.nlm.nih.gov/, SRR11496386 https://www.ncbi.nlm.nih.gov/, SRR11496387.

## Author Contributions

XL and SL conceived and designed the research. XL and MZ performed the research. ZX and MZ analyzed the data. XL wrote the manuscript. SL and HX revised the manuscript. All authors contributed to the article and approved the submitted version.

## Conflict of Interest

The authors declare that the research was conducted in the absence of any commercial or financial relationships that could be construed as a potential conflict of interest.
