# Supplementary material for: Construction of a Robust Sphingomonas sp. Strain for Welan Gum Production via the Expression of Global Transcriptional Regulator IrrE
Source: Front Bioeng Biotechnol. 2020 Jun 30;8:674. doi: 10.3389/fbioe.2020.00674 (PMC7338795; doi:10.3389/fbioe.2020.00674)
Supplement: Supplementary file 1 [file Data_Sheet_1.pdf]

Supplementary

Table 1 Primers used in this study

Name	Sequence (5’-3’)
**Primers for fragments that connect pet-28a**	
pET-*groes*-F	TAAGAAGGAGATATACCATGGGCATGAGACTAAAGCCA
pET-*groes*-R	TGGTGGTGCTCGAGTGCGGCCGCTTATTCGATTATTGCTAG
pET-*groel*- F	GAAGGAGATATACCATGGGCATGGCGAAGATCCTG
pET-*groel*- R	TGGTGGTGCTCGAGTGCGGCCGCTTAGAAGTCCATGTCCCC
pET-*irre*- F	TAAGAAGGAGATATACCATGGCCATGCCCAGTGCCAACGTC
pET-*irre*- R	TGGTGGTGCTCGAGTGCGGCCGCTCACTGTGCAGCGTCCTG
**Primers for fragments that connect pBBR1MCS-5**	
pBBR-*groes*- F	GGTATCGATAAGCTTTAATACGACTCACTA
pBBR-*groes*- R	AGAACTAGTGGATCCTTATTCGATTATTGC
pBBR-*groel*- F	GGTATCGATAAGCTTTAATACGACTCACTA
pBBR-*groel*- R	AGAACTAGTGGATCCTTAATTAGAAGTCCATGTC
pBBR-*irre*- F	GGTATCGATAAGCTTTAATACGACTCACTA
pBBR-*irre*- R	AGAACTAGTGGATCCTCACTGTGCAGCGTC
**Degenerate primers for key enzyme genes of carotenoid synthesis pathway amplification**	
crtB-F	ATGCGCTTTCCCGGCCGCGAY
crtB-R	TYAKCGCGCCCGSGTCCASAG
crtG-F	ATGRACRTCGCSCTTGCCWTS
crtG-R	TCAAKCTCCAGCGTCGCCAGY
crtY-F	ATGCGCCGCGCAGYCGTGATC
crtY-R	TCASRCCAWYTCGCCCAGCAT
crtI-F	ATGCCTTCAACCATCAGTTGY
crtI-R	TCAGCGGYTCCAGATCGCRGA

crtZ-F	ATGTCSCYSCYMAMYGSMWTY
crtZ-R	TCAATCCGGGATSGTSRTSSR
**Primers for *crtB* deletion**	
crtB-L-F	TTCCTGCAGCCCGGGGGATCCCACTATGCGCCCACCGACTT
crtB-L-R	GCGTCCACAGTCCTGCCGTGACCGTCGGCGATATCGT
crtB-R-F	ACGATATCGCCGACGGTCACGGCAGGACTGTGGACGC
crtB-R-R	ACCGCGGTGGCGGCCGCTCTAGAGCTGGTGAGCGGCGCTAA
Gm-F	ATGTTACGCAGCAGCAAC
Gm-R	CGAATTGTTAGGTGGCGG
**Primers for *irre* insertion**	
crtB-L-F	TTCCTGCAGCCCGGGGGATCCCACTATGCGCCCACCGACTT
crtB-L-R	TAGTGAGTCGTATTAGACCGTCGGCGATATCGT
crtB-R-F	CAAGATGCCGCGCAGTGACGGTAGGACTGTGGACGC
crtB-R-R	ACCGCGGTGGCGGCCGCTCTAGAGCTGGTGAGCGGCGCTAA
irre-△*crtB*-F	ACGATATCGCCGACGGTCTAATACGACTCACTA
irre-△*crtB*-R	GCGTCCACAGTCCTACCGTCACTGCGCGGCATCTTG

Fig.1 Plasmid construction strategy

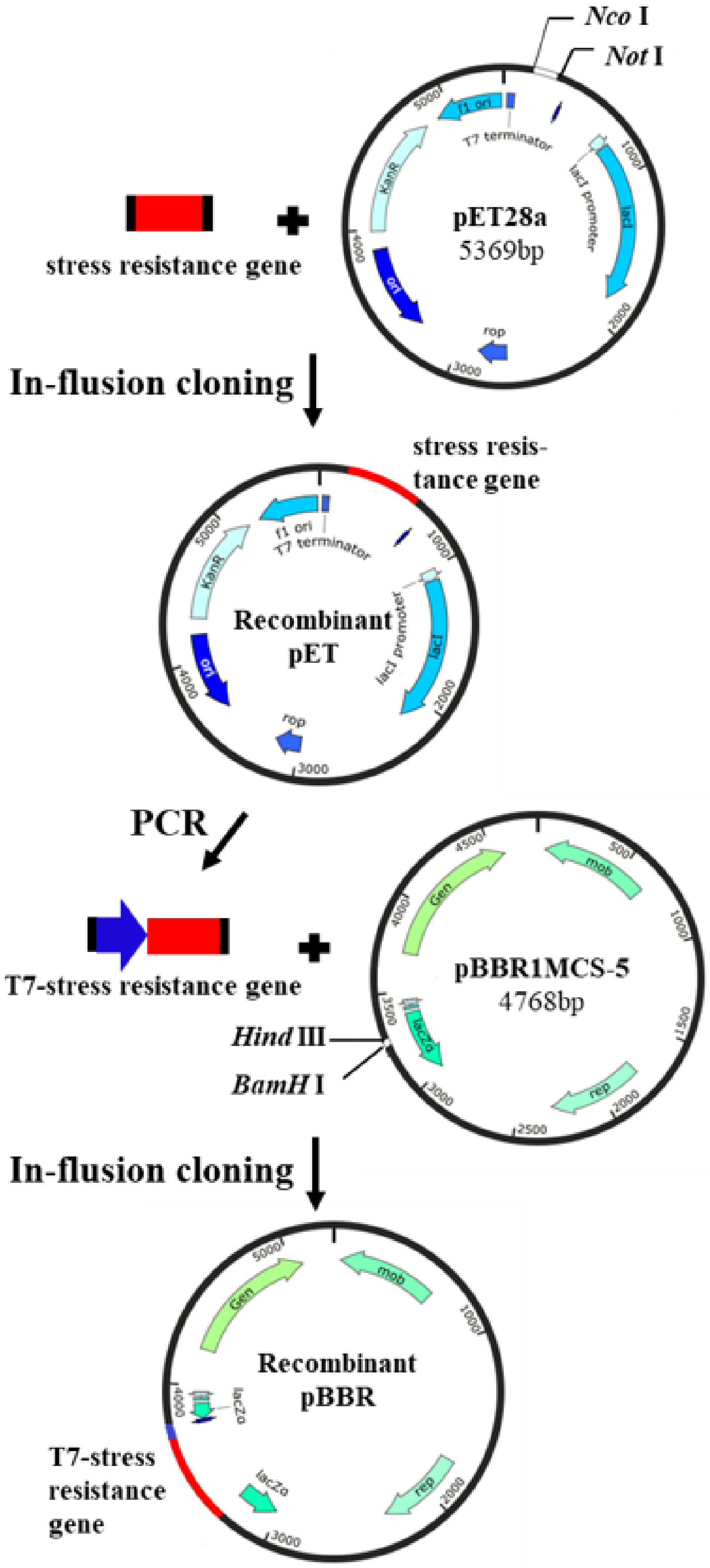

Fig. 2 Gene deletion strategy

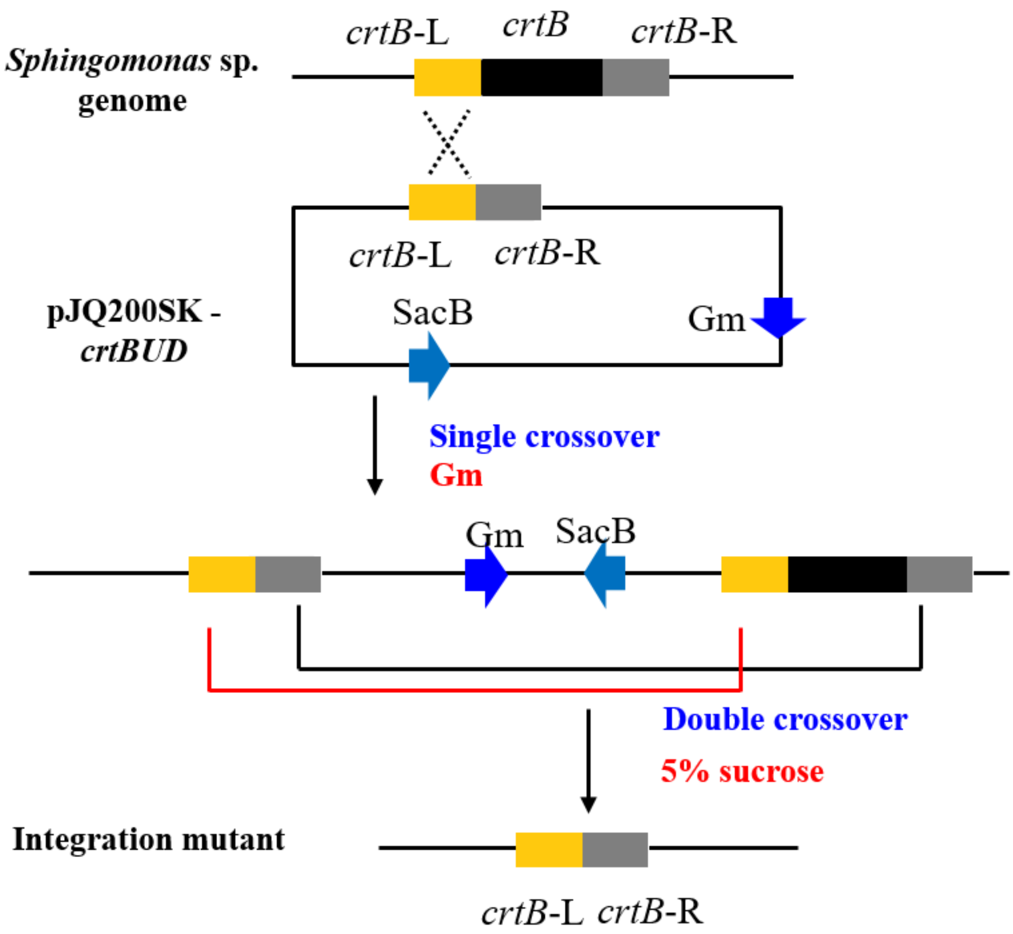

Fig. 3 Effects of *crtB* deletion on product color

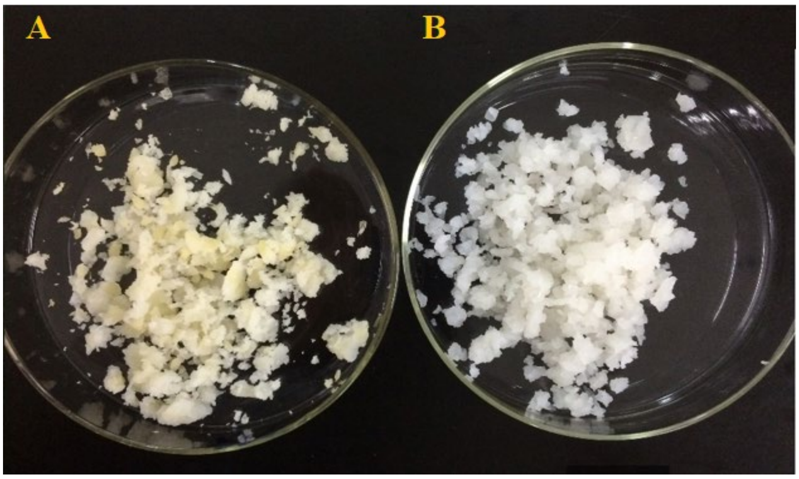

A. Product of *S*. sp. NX-3. B. Product of *S*. sp.-*Δcrt*B.
